# Supplementary material for: Differential transcript usage unravels gene expression alterations in Alzheimer’s disease human brains
Source: NPJ Aging Mech Dis. 2021 Jan 4;7:2. doi: 10.1038/s41514-020-00052-5 (PMC7782705; doi:10.1038/s41514-020-00052-5)
Supplement: Supplementary file 1 — Supplementary Material [file 41514_2020_52_MOESM1_ESM.pdf]

Differential transcript usage unravels gene expression alterations in Alzheimer's disease human brains

Diego Marques-Coelho[1,2], Lukas Iohan da Cruz Carvalho[1,2], Ana Raquel Melo de Farias[1,3], Amandine Flaig[3], The Brainbank Neuro-CEB Neuropathology Network[4], Jean-Charles Lambert[3], Marcos Romualdo Costa[1,3,*]

Supplementary Figures:

Supplementary Figure 1. Allen Brain Atlas scRNAseq MTG dataset information. A) tSNE representation showing distribution of single cells in two dimensions; B) Donut plot explaining percentage of cell types among all groups with absolute number of cells; C) Dotplot showing cell type known markers elucidating cell clusters findings; D) Boxplot demonstrating distribution of average expression among all cell types; E) UpsetR exhibiting number of genes expressed in each cell type combination.

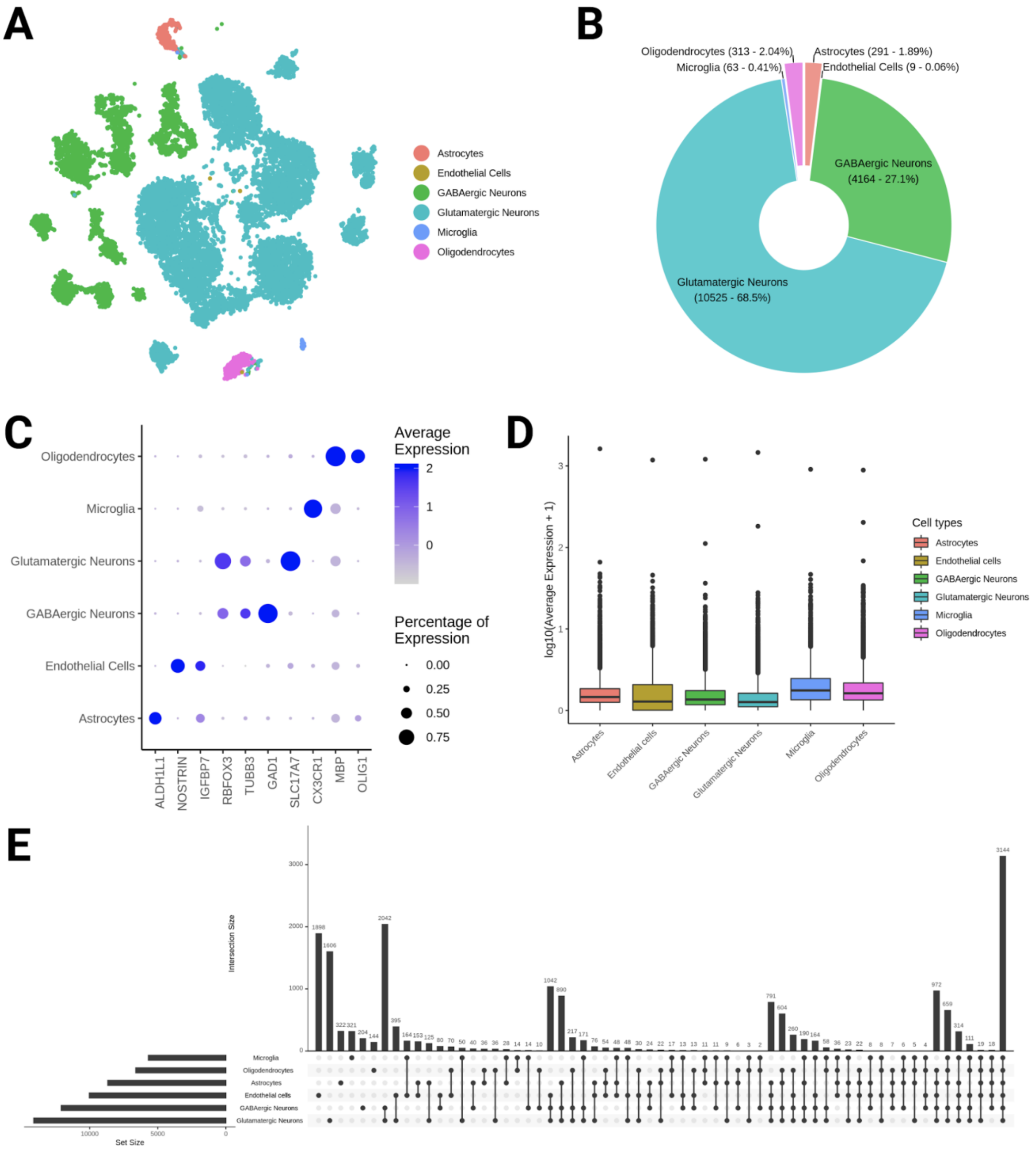
A
Astrocytes
Endothelial Cells
GABAergic Neurons
Glutamatergic Neurons
Microglia
Oligodendrocytes
B
Oligodendrocytes (313 - 2.04%)
Microglia (63 - 0.41%)
Astrocytes (291 - 1.89%)
Endothelial Cells (9 - 0.06%)
GABAergic Neurons (4164 - 27.1%)
Glutamatergic Neurons (10525 - 68.5%)
C
Average Expression
2
1
0
Percentage of Expression
0.00
0.25
0.50
0.75
Oligodendrocytes
Microglia
Glutamatergic Neurons
GABAergic Neurons
Endothelial Cells
Astrocytes
ALDH1L1
NOSTRIN
IGFBP7
RBFOX3
TUBB3
GAD1
SLC17A7
CX3CR1
MBP
OLIG1
D
log10(Average Expression + 1)
Cell types
Astrocytes
Endothelial cells
GABAergic Neurons
Glutamatergic Neurons
Microglia
Oligodendrocytes
E
Intersection Size
Set Size
Microglia
Oligodendrocytes
Astrocytes
Endothelial cells
GABAergic Neurons
Glutamatergic Neurons

Supplementary Figure 2. scRNAseq signatures comparison with Grubman and Mathys et al works in Glutamatergic Neurons, GABAergic Neurons and Astrocytes. A,C,E) UpsetR and Venn Diagram illustrate intersection among different studies; B,D,F) Gene-set enrichment analysis (GSEA) with cell-type signatures among all studies. As a threshold for GSEA, FDR < 0.01, intersection size > 3 and precision > 0.05. Analyses were made for glutamatergic neurons (A-B), gabaergic neurons (C-D) and astrocytes (E-F).

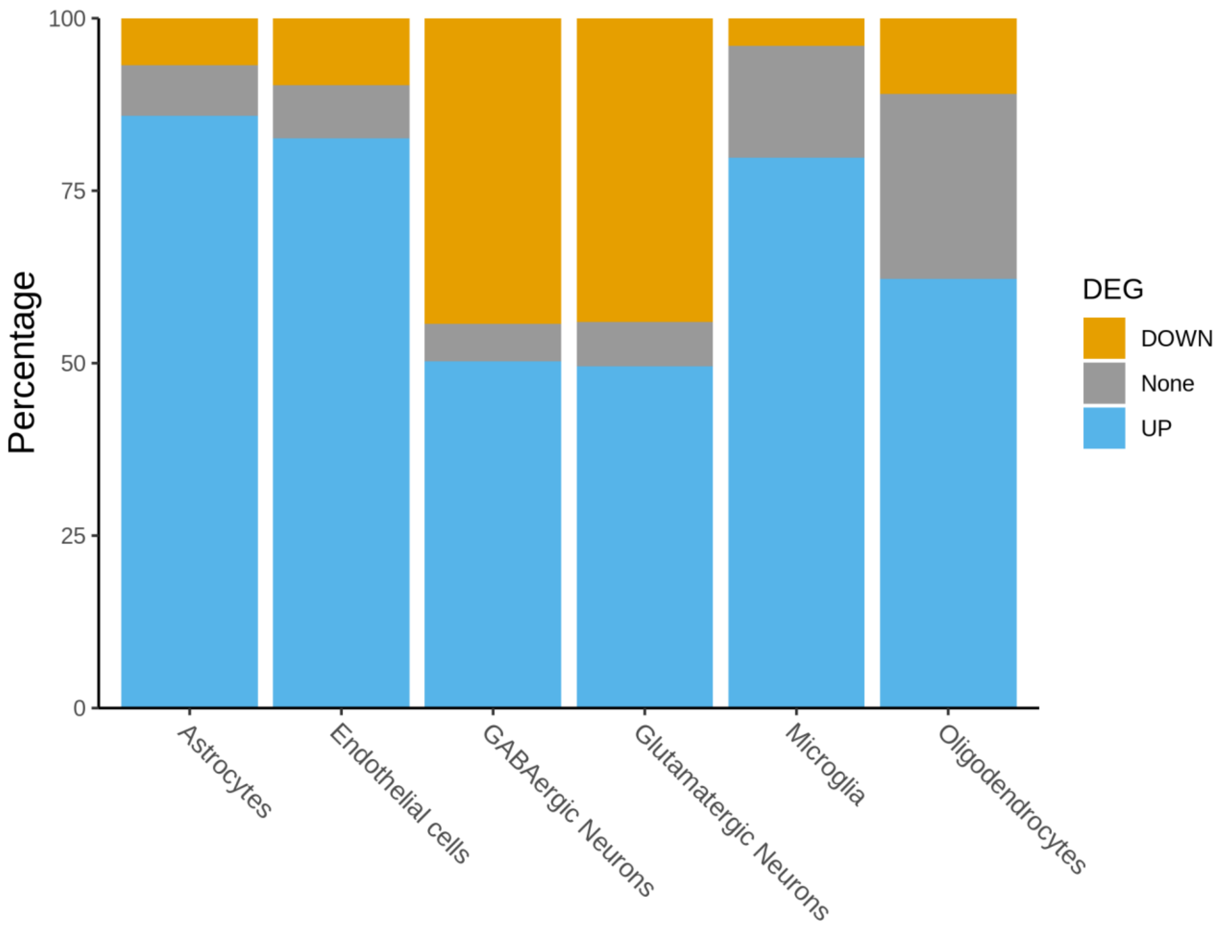

Supplementary Figure 3. Percentage of DEGs regulation among cell types. Graphic showing the percentage of genes UP (blue) and DOWN (yellow) regulated in both TL datasets (Mayo and MSBB_TL), as well as genes altered in discordant directions in these two datasets (grey). Observe that neurons have about half of the genes altered in each direction, whereas astrocytes, oligodendrocytes, microglia and endothelial cells have a predominance of up regulated genes.

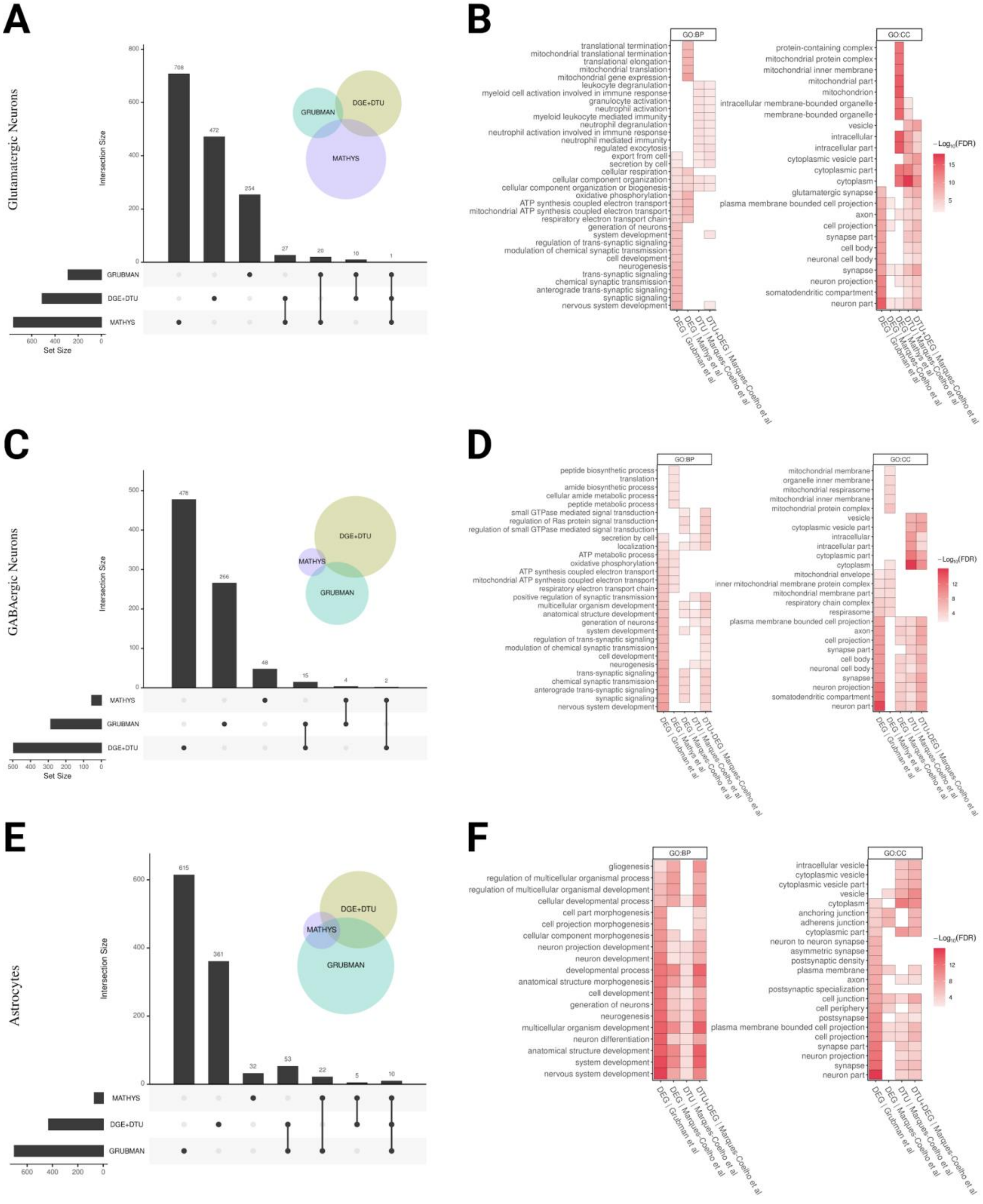

Supplementary Figure 4. scRNAseq signatures comparison with Grubman and Mathys et al works in Oligodendrocytes, Endothelial cells and Microglia. A,C,E) Upset plot and

Venn Diagram illustrate intersection among different studies; B,D,F) Gene-set enrichment analysis (GSEA) with cell-type signatures among all studies. As a threshold for GSEA, FDR < 0.01, intersection size > 3 and precision > 0.05. Analyses were made for oligodendrocytes (A-B), endothelial cells (C-D) and microglia (E-F).

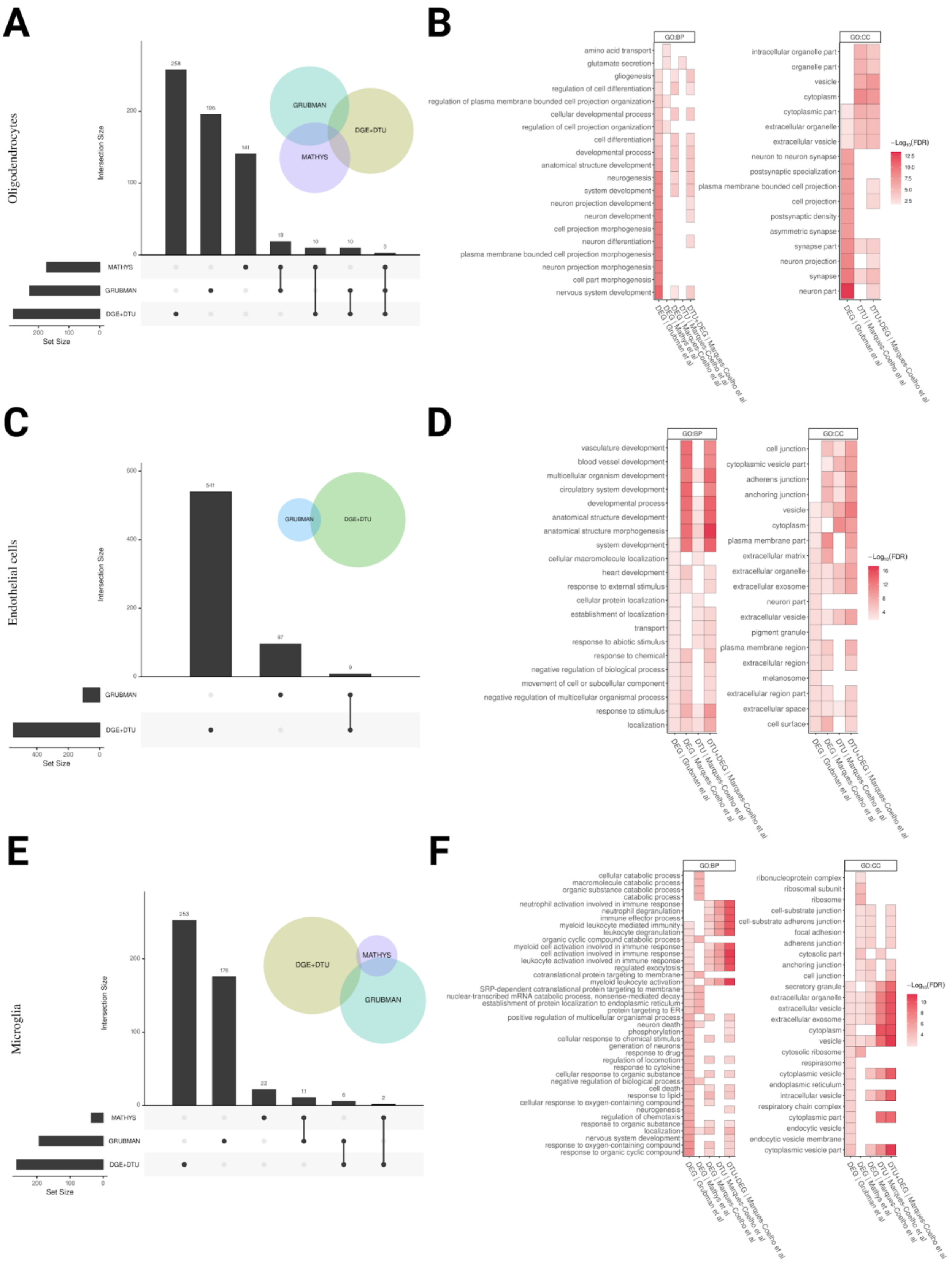

A
Oligodendrocytes
Intersection Size
258
196
141
19
10
10
3
GRUBMAN
DGE+DTU
MATHYS
Set Size
B
GO:BP
amino acid transport
glutamate secretion
gliogenesis
regulation of cell differentiation
regulation of plasma membrane bounded cell projection organization
cellular developmental process
regulation of cell projection organization
cell differentiation
developmental process
anatomical structure development
neurogenesis
system development
neuron projection development
neuron development
cell projection morphogenesis
neuron differentiation
plasma membrane bounded cell projection morphogenesis
neuron projection morphogenesis
cell part morphogenesis
nervous system development
DEG | Grubman et al
DEG | Mathys et al
DEG | Marques-Coelho et al
DTU | Marques-Coelho et al
DTU+DEG | Marques-Coelho et al
GO:CC
intracellular organelle part
organelle part
vesicle
cytoplasm
cytoplasmic part
extracellular organelle
extracellular vesicle
neuron to neuron synapse
postsynaptic specialization
plasma membrane bounded cell projection
cell projection
postsynaptic density
asymmetric synapse
synapse part
neuron projection
synapse
neuron part
DEG | Grubman et al
DTU | Marques-Coelho et al
DTU+DEG | Marques-Coelho et al
−Log10(FDR)
12.5
10.0
7.5
5.0
2.5
C
Endothelial cells
Intersection Size
541
97
9
GRUBMAN
DGE+DTU
Set Size
D
GO:BP
vasculature development
blood vessel development
multicellular organism development
circulatory system development
developmental process
anatomical structure development
anatomical structure morphogenesis
system development
cellular macromolecule localization
heart development
response to external stimulus
cellular protein localization
establishment of localization
transport
response to abiotic stimulus
response to chemical
negative regulation of biological process
movement of cell or subcellular component
negative regulation of multicellular organismal process
response to stimulus
localization
DEG | Grubman et al
DEG | Marques-Coelho et al
DTU | Marques-Coelho et al
DTU+DEG | Marques-Coelho et al
GO:CC
cell junction
cytoplasmic vesicle part
adherens junction
anchoring junction
vesicle
cytoplasm
plasma membrane part
extracellular matrix
extracellular organelle
extracellular exosome
neuron part
extracellular vesicle
pigment granule
plasma membrane region
extracellular region
melanosome
extracellular region part
extracellular space
cell surface
DEG | Grubman et al
DEG | Marques-Coelho et al
DTU | Marques-Coelho et al
DTU+DEG | Marques-Coelho et al
−Log10(FDR)
16
12
8
4
E
Microglia
Intersection Size
253
176
22
11
6
2
DGE+DTU
MATHYS
GRUBMAN
Set Size
F
GO:BP
cellular catabolic process
macromolecule catabolic process
organic substance catabolic process
catabolic process
neutrophil activation involved in immune response
neutrophil degranulation
immune effector process
myeloid leukocyte mediated immunity
leukocyte degranulation
organic cyclic compound catabolic process
myeloid cell activation involved in immune response
cell activation involved in immune response
leukocyte activation involved in immune response
regulated exocytosis
cotranslational protein targeting to membrane
myeloid leukocyte activation
SRP-dependent cotranslational protein targeting to membrane
nuclear-transcribed mRNA catabolic process, nonsense-mediated decay
establishment of protein localization to endoplasmic reticulum
protein targeting to ER
positive regulation of multicellular organismal process
neuron death
phosphorylation
cellular response to chemical stimulus
generation of neurons
response to drug
regulation of locomotion
response to cytokine
cellular response to organic substance
negative regulation of biological process
cell death
response to lipid
cellular response to oxygen-containing compound
neurogenesis
regulation of chemotaxis
response to organic substance
localization
nervous system development
response to oxygen-containing compound
response to organic cyclic compound
DEG | Grubman et al
DEG | Mathys et al
DEG | Marques-Coelho et al
DTU | Marques-Coelho et al
DTU+DEG | Marques-Coelho et al
GO:CC
ribonucleoprotein complex
ribosomal subunit
ribosome
cell-substrate junction
cell-substrate adherens junction
focal adhesion
adherens junction
cytosolic part
anchoring junction
cell junction
secretory granule
extracellular organelle
extracellular vesicle
extracellular exosome
cytoplasm
vesicle
cytosolic ribosome
respirasome
cytoplasmic vesicle
endoplasmic reticulum
intracellular vesicle
respiratory chain complex
cytoplasmic part
endocytic vesicle
endocytic vesicle membrane
cytoplasmic vesicle part
DEG | Grubman et al
DEG | Mathys et al
DEG | Marques-Coelho et al
DTU | Marques-Coelho et al
DTU+DEG | Marques-Coelho et al
−Log10(FDR)
10
8
6
4

Supplementary Figure 5. Expression of BIN1 isoform 1 is reduced in AD brains. A) Western blot for BIN1 and ACTIN in brain lysates obtained from the frontal cortex (FCx) and hippocampus (Hip) of non-pathology (n=3) and AD (n=6) individuals. B-C) Quantification of total BIN1 normalized per ACTIN (B) and BIN1 isoform 1 normalized by the total BIN1 (C). ANOVA $F_{(3,14)}$= 3.36 and p=0.0494 (B); $F_{(3,14)}$= 16.24 and p<0.0001 (C); Tukey's multiple comparisons test * Padj<0.05 and *** Padj<0.001. D) Western blot for BIN1 in brain lysates from 3 controls and 6 AD individuals (Hip and FCx samples are intercalated) and HEK cells transfected with lentiviral vectors carrying plasmids encoding for BIN1 isoforms 1 and 9. Observe the correspondence between the heavy (~80 KDa) and light (~60 KDa) bands in the brain with those observed after expression of BIN1 isoforms 1 and 9, respectively.

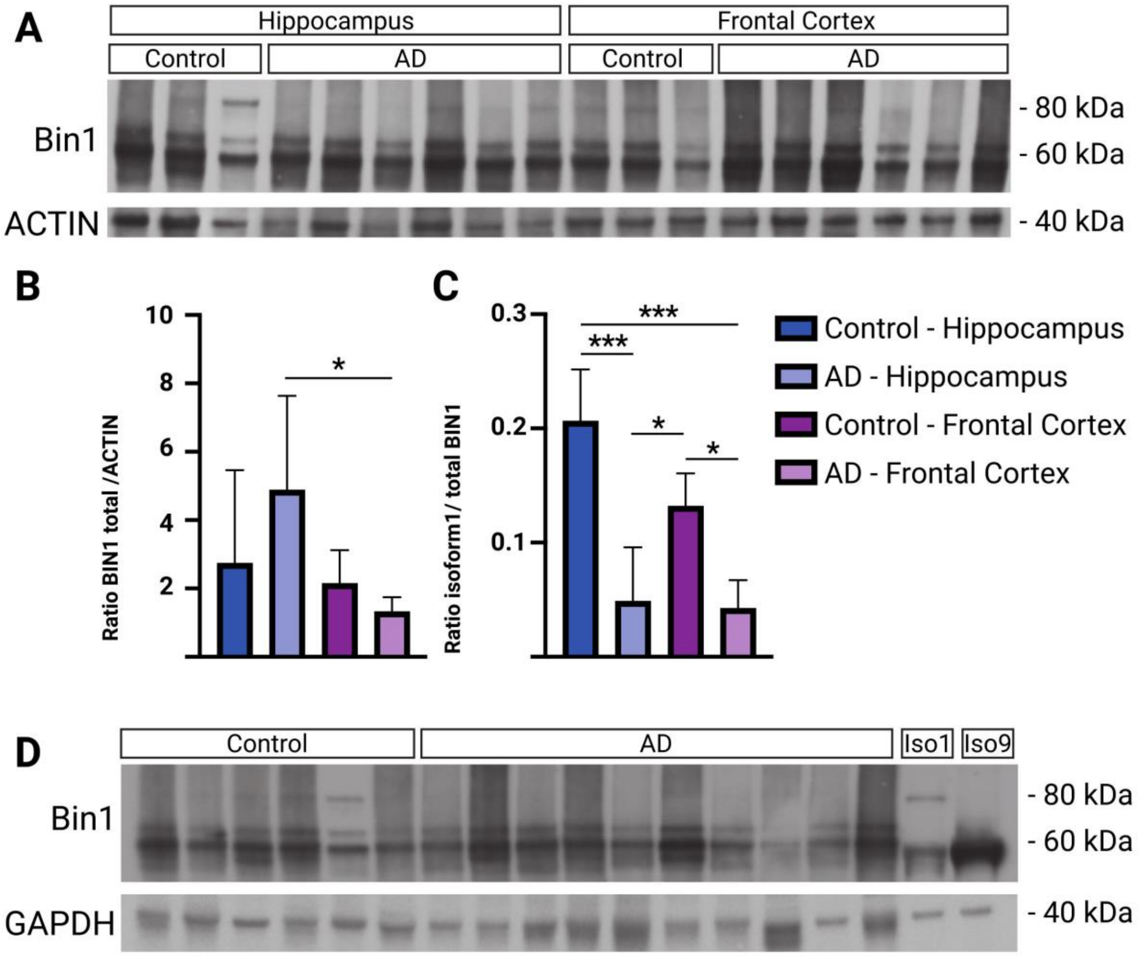

Description of supplementary tables:

Supplementary table 1. Summary statistics for DEG and DTU analyses

Supplementary table 2. List of DEGs and gDTUs in TLI and FLI

Supplementary table 3. GSEA for DEGs and gDTUs in the temporal lobe intersection (TLI)

Supplementary table 4. Summary of splicing events (SE) and events consequences (EC) observed in different datasets

Supplementary table 5. Summary statistics for DTU analysis in MSBB using Braak stages

Supplementary table 6. List of splicing related genes used in this study and their corresponding ontology terms

Supplementary table 7. Expression of genes identified in DEGs and DTU analyses in different cell types/subtypes

Supplementary table 8. GSEA for DEGs and gDTUs in the TLI per cell type

Supplementary table 9. GSEA for DEGs identified in different cell types in previous single-cell RNAseq studies

Supplementary table 10. List of AD risk genes used in this study

Supplementary table 11. Expression of AD risk/causal factors in different cell types/subtypes

S**upplementary table 12.** Summary of clinico-pathological criteria used in different consortia to classify AD and control subjects

Supplementary table 13. Metadata for all individual samples used in this study
