# Supplementary material for: Differential transcript usage unravels gene expression alterations in Alzheimer’s disease human brains
Source: NPJ Aging Mech Dis. 2021 Jan 4;7:2. doi: 10.1038/s41514-020-00052-5 (PMC7782705; doi:10.1038/s41514-020-00052-5)
Supplement: Supplementary file 15 — reporting summary [file 41514_2020_52_MOESM15_ESM.pdf]

nature research

Corresponding author(s): Marcos R. Costa

Last updated by author(s): Aug 18, 2020

Reporting Summary

Nature Research wishes to improve the reproducibility of the work that we publish. This form provides structure for consistency and transparency in reporting. For further information on Nature Research policies, see our Editorial Policies and the Editorial Policy Checklist.

Statistics

For all statistical analyses, confirm that the following items are present in the figure legend, table legend, main text, or Methods section.

n/a	Confirmed	
☐	☒	The exact sample size (*n*) for each experimental group/condition, given as a discrete number and unit of measurement
☐	☒	A statement on whether measurements were taken from distinct samples or whether the same sample was measured repeatedly
☐	☒	The statistical test(s) used AND whether they are one- or two-sided<br>*Only common tests should be described solely by name; describe more complex techniques in the Methods section.*
☐	☒	A description of all covariates tested
☐	☒	A description of any assumptions or corrections, such as tests of normality and adjustment for multiple comparisons
☐	☒	A full description of the statistical parameters including central tendency (e.g. means) or other basic estimates (e.g. regression coefficient) AND variation (e.g. standard deviation) or associated estimates of uncertainty (e.g. confidence intervals)
☐	☒	For null hypothesis testing, the test statistic (e.g. F, t, r) with confidence intervals, effect sizes, degrees of freedom and P value noted<br>*Give P values as exact values whenever suitable.*
☐	☒	For Bayesian analysis, information on the choice of priors and Markov chain Monte Carlo settings
☐	☒	For hierarchical and complex designs, identification of the appropriate level for tests and full reporting of outcomes
☐	☒	Estimates of effect sizes (e.g. Cohen's d, Pearson's r), indicating how they were calculated

Our web collection on statistics for biologists contains articles on many of the points above.

Software and code

Policy information about availability of computer code

Data collection	MSBB RNAseq (De Jager et al, 2018) - https://doi.org/10.7303/syn3388564<br>ROSMAP RNAseq (Wang et al, 2018) - https://doi.org/10.7303/syn3157743<br>MAYO RNAseq (Allen et al, 2018) - https://doi.org/10.7303/syn5550404
Data analysis	synapseclient 1.9.1 (Sage Bionetworks) - https://python-docs.synapse.org/build/html/index.html<br>kallisto 0.43.1 (Bray et al, 2016) - https://pachterlab.github.io/kallisto/about<br>tximport 1.12.3 (Soneson et al, 2015) - https://www.bioconductor.org/packages/release/bioc/html/tximport.html<br>DESeq2 1.24.0 (Love et al, 2014) - https://www.bioconductor.org/packages/release/bioc/html/DESeq2.html<br>IsoformSwitchAnalyzeR 1.6.0 (Vitting-Seerup et al, 2017) - https://www.bioconductor.org/packages/release/bioc/html/IsoformSwitchAnalyzeR.html<br>DRIMseq 1.12.0 (Nowicka et al, 2016) - https://www.bioconductor.org/packages/release/bioc/html/DRIMSeq.html<br>StageR 1.6.0 (Van den Berge & Clement et al, 2019) - https://www.bioconductor.org/packages/release/bioc/html/stageR.html<br>Seurat 3.1.1 (Butler et al, 2019) - https://satijalab.org/seurat/<br>gprofiler2 0.1.9 (Kohlberg & Raudvere et al, 2020) - https://cran.r-project.org/web/packages/gprofiler2/index.html<br>ggplot2 3.3.2 (Wickham et al, 2016) - https://ggplot2.tidyverse.org/<br>R 3.6 - https://www.r-project.org/<br>Python 3.7 - https://www.python.org/

For manuscripts utilizing custom algorithms or software that are central to the research but not yet described in published literature, software must be made available to editors and reviewers. We strongly encourage code deposition in a community repository (e.g. GitHub). See the Nature Research guidelines for submitting code & software for further information.

Data

Policy information about availability of data

All manuscripts must include a data availability statement. This statement should provide the following information, where applicable:

- Accession codes, unique identifiers, or web links for publicly available datasets
- A list of figures that have associated raw data
- A description of any restrictions on data availability

Provide your data availability statement here.

Field-specific reporting

Please select the one below that is the best fit for your research. If you are not sure, read the appropriate sections before making your selection.

☒ Life sciences ☐ Behavioural & social sciences ☐ Ecological, evolutionary & environmental sciences

For a reference copy of the document with all sections, see nature.com/documents/nr-reporting-summary-flat.pdf

Life sciences study design

All studies must disclose on these points even when the disclosure is negative.

Sample size	We used publicly available datasets. Therefore, sample sizes were limited by the number of samples in the datasets No we just confirmed that they were large enough for the statistical tests used in our analyses.
Data exclusions	No data was excluded from the analyses.
Replication	Whenever possible, analyses were performed with different tools to confirm the findings.
Randomization	Allocation of samples was not random. "Post-mortem interval (PMI)" and "RNA integrity number (RIN)" were used s covariates in our model to control for possible “batch effects” (linear regression). "Age of death" and "Sex" were balanced among the different groups.
Blinding	Blinding was not relevant for our study, because analyses were performed using bioinformatic tools and do not rely on subjective evaluation.

Reporting for specific materials, systems and methods

We require information from authors about some types of materials, experimental systems and methods used in many studies. Here, indicate whether each material, system or method listed is relevant to your study. If you are not sure if a list item applies to your research, read the appropriate section before selecting a response.

Materials & experimental systems

n/a	Involved in the study	
☐	☒	Antibodies
☒	☐	Eukaryotic cell lines
☒	☐	Palaeontology and archaeology
☒	☐	Animals and other organisms
☒	☐	Human research participants
☒	☐	Clinical data
☒	☐	Dual use research of concern

Methods

n/a	Involved in the study	
☒	☐	ChIP-seq
☒	☐	Flow cytometry
☒	☐	MRI-based neuroimaging

Antibodies

Antibodies used	Anti-BIN1 clone EPR13463 - Abcam Cat# ab182562, Lot# GR3275123-2<br>Anti-GAPDH clone HEK293 - Merck millipore Cat# AB2302, Lot# 3088383<br>Anti-BetaActin clone AC15 - Sigma Aldrich Cat# 1978, source# 22190701, batch# 0000086304
Validation	Anti-BIN1 (Abcam, 182562) - KO validated. References: 1) Ge Y et al. The splicing factor RBM25 controls MYC activity in acute myeloid leukemia. Nat Commun 10:172 (2019); 2) De Rossi et al. Neuronal BIN1 Regulates Presynaptic Neurotransmitter Release and Memory Consolidation. Cell Reports 10:3520 (2020).
